# 5-FU Metabolism in Cancer and Orally-Administrable 5-FU Drugs

**DOI:** 10.3390/cancers2031717

**Published:** 2010-09-17

**Authors:** Koh Miura, Makoto Kinouchi, Kazuyuki Ishida, Wataru Fujibuchi, Takeshi Naitoh, Hitoshi Ogawa, Toshinori Ando, Nobuki Yazaki, Kazuhiro Watanabe, Sho Haneda, Chikashi Shibata, Iwao Sasaki

**Affiliations:** 1 Department of Surgery, Tohoku University Graduate School of Medicine, Sendai, Japan; E-Mails: kinouchi@surg1.med.tohoku.ac.jp (M.K.); hogawa@surg1.med.tohoku.ac.jp (H.O.); ando@surg1.med.tohoku.ac.jp (T.A.); n_yazaki@surg1.med.tohoku.ac.jp (N.Y.); k-wata@surg1.med.tohoku.ac.jp (K.W.); sho@surg1.med.tohoku.ac.jp (S.H.); cshibata@surg1.med.tohoku.ac.jp (C.S.); isasaki@surg1.med.tohoku.ac.jp (I.S.); 2 Department of Pathology, Tohoku University Hospital, Sendai, Japan; E-Mail: musubi@patholo2.med.tohoku.ac.jp; 3 Computational Biology Research Center, National Institute of Advanced Industrial Science and Technology, Tokyo, Japan; E-Mail: w.fujibuchi@aist.go.jp

**Keywords:** 5-FU metabolism, cell death, colon cancer, oral 5-FU drugs

## Abstract

5-Fluorouracil (5-FU) is a key anticancer drug that for its broad antitumor activity, as well as for its synergism with other anticancer drugs, has been used to treat various types of malignancies. In chemotherapeutic regimens, 5-FU has been combined with oxaliplatin, irinotecan and other drugs as a continuous intravenous infusion. Recent clinical chemotherapy studies have shown that several of the regimens with oral 5-FU drugs are not inferior compared to those involving continuous 5-FU infusion chemotherapy, and it is probable that in some regimens continuous 5-FU infusion can be replaced by oral 5-FU drugs. Historically, both the pharmaceutical industry and academia in Japan have been involved in the development of oral 5-FU drugs, and this review will focus on the current knowledge of 5-FU anabolism and catabolism, and the available information about the various orally-administrable 5-FU drugs, including UFT, S-1 and capecitabine. Clinical studies comparing the efficacy and adverse events of S-1 and capecitabine have been reported, and the accumulated results should be utilized to optimize the treatment of cancer patients. On the other hand, it is essential to elucidate the pharmacokinetic mechanism of each of the newly-developed drugs, to correctly select the drugs for each patient in the clinical setting, and to further develop optimized drug derivatives.

## 1. Introduction

Since its introduction more than 50 years ago, 5-fluorouracil (5-FU) has become a key anticancer drug that has been used to treat various types of malignancies for its broad antitumor activity, as well as its synergism with other anticancer drugs. In 1957, Heidelberger *et al*. [1] reported the development of 5-FU, but several important findings had preceded their work. For example, in 1954 Rutman *et al*. [2] showed that uracil was incorporated into rat hepatomas more rapidly than normal tissues; and in 1956 Handschumacher *et al*. reported the tumor-inhibitory activity by 6-azauracil [3]. In recent chemotherapeutic regimens, the continuous intravenous infusion of 5-FU has been combined with oxaliplatin, irinotecan and other drugs. The continuous 5-FU infusion is based on an official report published in the US in 1964 [4], showing that 5-FU is a time-dependent antimetabolite. The meta-analysis of more than 1,200 colorectal cancer patients in six randomized clinical trials, which showed the efficacy of continuous 5-FU infusion compared with bolus 5-FU administration [5], also supported the importance of continuous 5-FU infusion. Based on these results, continuous 5-FU infusion regimens, such as FOLFOX or FOLFIRI, have been established and are widely utilized. On the other hand, recent clinical studies have shown that several of the chemotherapeutic regimens with oral 5-FU drugs are not inferior to those with continuous 5-FU infusion chemotherapy, and in some regimens it may be possible to replace continuous 5-FU infusion chemotherapies with oral 5-FU drugs. Historically, both the pharmaceutical industry and academia in Japan have contributed to the development of oral 5-FU drugs. This review will summarize the current knowledge about 5-FU metabolism, and the information about orally-administrable 5-FU drugs.

## 2. 5-FU Metabolism

**Figure 1:**
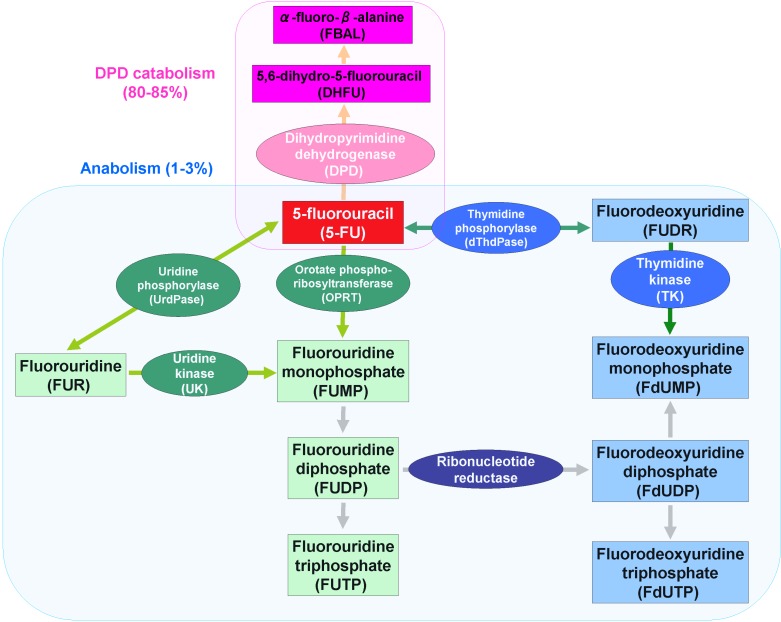
5-FU anabolism and catabolism.

It has been demonstrated that 80% to 85% of 5-FU is catabolized to inactive metabolites by dihydropyrimidine dehydrogenase (DPD), and only 1 to 3% of the original dose of 5-FU mediates the cytotoxic effects on tumor cells and normal tissues through anabolic actions [6], thereby inhibiting DNA synthesis and RNA processing and function (Figure 1). The 5-FU metabolite, fluorodeoxyuridine monophosphate (FdUMP), forms a ternary complex with thymidylate synthase (TS) and 5,10-methylene tetrahydrofolate (CH2THF), thereby inhibiting the synthesis of DNA.

### 2.1. 5-FU Anabolism

The chemotherapeutic compound 5-FU is a uracil analogue with a fluorine atom at the C-5 position. After intravenous administration of 5-FU, it rapidly enters cells using the same transport mechanism as uracil [7]. The processing mechanism of 5-FU in cells is as diverse as that of normal pyrimidines, and the current understanding of the metabolism is summarized in Figure 1. First, 5-FU is converted to the following active metabolites: 1) fluorouridine triphosphate (FUTP), which is incorporated into RNA instead of uridine triphosphate (UTP); 2) fluorodeoxyuridine triphosphate (FdUTP), which is incorporated into DNA instead of deoxythymidine triphosphate (dTTP); and 3) FdUMP, which inhibits the activity of TS in the ternary complex, as described in the previous section. FUTP causes alterations in RNA processing and function, and FdUTP and FdUMP cause DNA damage; both of these processes affect RNA and DNA and cause cell death.

As mentioned, a US report published in 1964 demonstrated 5-FU to be a time-dependent antimetabolite [4]. The main mechanism of 5-FU activation is conversion to fluorouridine monophosphate (FUMP), either directly by orotate phosphoribosyltransferase (OPRT) with phosphoribosyl pyrophosphate as a cofactor, or indirectly via fluorouridine (FUR) through the sequential action of uridine phosphorylase (UrdPase) and uridine kinase (UK) [8]. The other 5-FU activation pathway involves thymidine phosphorylase (dThdPase), which catalyzes the conversion of 5-FU to fluorodeoxyuridine (FUDR), and FUDR is then phosphorylated by thymidine kinase (TK) to FdUMP. In this series of reactions, the phosphorylation reaction by the UrdPase requires ribose-1-phosphate as a cofactor, eventually synthesizing FUMP. In contrast, the phosphorylation reaction by dThdPase requires deoxyribose-1-phosphate as a cofactor, eventually leading to the synthesis of FdUMP. FUMP is further phosphorylated to fluorouridine diphosphate (FUDP), which is either further phosphorylated to the active metabolite FUTP, or converted to fluorodeoxyuridine diphosphate (FdUDP) by ribonucleotide reductase [8]. FdUDP is then either further phosphorylated to FdUTP, or dephosphorylated to FdUMP. Both FdUTP and FdUMP cause DNA damage. 

The conversion of 5-FU to FdUMP in the gastrointestinal (GI) tract and bone marrow elicits GI toxicity and myelotoxicity, respectively. In 1979, an *in vivo* mouse study by Houghton *et al*. indicated that GI toxicity was caused by the incorporation of fluorinated pyrimidines, mainly FdUMP [9]. In 1984, Schuetz *et al*. analyzed the myelotoxicity of 5-FU using CF-1 mouse bone marrow cells under 5-FU exposure *in vitro* [10], and demonstrated that 5-FU incorporation into DNA was closely associated with toxicity and inhibition of DNA synthesis with FdUMP [10]. Interestingly, the meta-analysis of six randomized clinical trials performed in 1998 showed that the grade 3 or 4 hematologic toxicity was more frequent in patients assigned to bolus 5-FU infusion rather than in those assigned to continuous 5-FU infusion [11].

### 2.2. 5-FU Catabolism

DPD is an enzyme present in the liver, intestinal mucosa and various other tissues. DPD catabolizes 5-FU to 5,6-dihydro-5-fluorouracil (DHFU) [12], finally leading to the formation of α-fluoro-β-ureido-propionic acid and α-fluoro-β-alanine (FBAL) (Figure 1). In 1987, Heggie *et al*. investigated the kinetics of 5-FU and 5-FU metabolites in cancer patients following intravenous bolus administration of radio-labeled 5-FU [13], and revealed that approximately 60–90% of the administered 5-FU was excreted in urine as FBAL within 24 hours. While most patients tolerate 5-FU reasonably well, a number of cancer patients with DPD deficiency were shown to be at increased risk for severe toxicities, including diarrhea, mucositis, and neurotoxicity, as well as death, after administration of standard doses of 5-FU [6].

Since the 1970s, the neurotoxicity of FBAL as a 5-FU catabolite has been discussed quite extensively [14,15]. Okeda *et al*. investigated the mechanism of 5-FU neurotoxicity with *in vivo* experiments using cats [15]. The two 5-FU metabolites, monofluoroacetic acid and FBAL, were continuously administered into the left ventricle of the brain in cats. In their experiments, two types of neuropathological changes, vacuoles and necrosis/softening-like changes, were detected, and FBAL was more toxic than monofluoroacetic acid. Both of the neuropathological changes in the FBAL group were similar to those found in patients following orally-administered 5-FU [15]. 

The cardiotoxicity of 5-FU has also been attributed to FBAL. Matsubara *et al*. investigated the mechanism of cardiotoxicity for 5-FU and its derivatives using *in vivo* experiments with anesthetized open-chest guinea pigs [16], and proposed that the formation of fluoroacetate, an inhibitor of aconitase, from 5-FU via FBAL, caused cardiotoxicity during chemotherapy [16]. As described in later publications, FBAL is also the main cause of hand-foot syndrome (HFS) acquired in cancer patients during 5-FU-based chemotherapy. In the 1998 meta-analysis HFS was more frequent in the continuous 5-FU infusion group than in the bolus 5-FU infusion group [5].

### 2.3. Ternary Complex

FdUMP forms a stable ternary complex with TS and CH2THF [17]. TS catalyzes the reductive methylation of deoxyuridine monophosphate (dUMP) to deoxythymidine monophosphate (dTMP) with the reduced folate CH2THF. The ternary complex blocks the access of dUMP to the nucleotide-binding site of TS by competition with FdUMP, which results in pool imbalances of deoxynucleotides, especially an increased level of deoxyuridine triphosphate (dUTP); leading to DNA damage. Depletion of dTMP results in the subsequent depletion of dTTP, which perturbs the levels of the other deoxynucleotides [18]. The pool imbalances of deoxynucleotides severely disrupt DNA synthesis and repair, again resulting in DNA damage [19]. As a result, the inhibition of TS results in the accumulation of dUMP, which leads to the increased levels of dUTP [20]. Thymidylate can be salvaged from thymidine through the action of TK, and this salvage pathway can also represent a mechanism of resistance to 5-FU [21]. Despite this information about the activity of 5-FU, the molecular mechanisms downstream of TS inhibition still have to be confirmed [8]. In addition, the clinical significance of TS needs to be demonstrated. In 2008, Showalter *et al*. investigated the connection between TS expression and 5-FU with a thorough literature survey, and in contrast to previous predictions, they found no connection between TS and the patient response to 5-FU [22]. To discuss this matter, we must remember that the influence of TS activity on 5-FU metabolism may change depending on the administration routes of 5-FU drugs, types of 5-FU drugs, the effects of LV, and other factors.

## 3. Oral 5-FU Drugs

**Figure 2:**
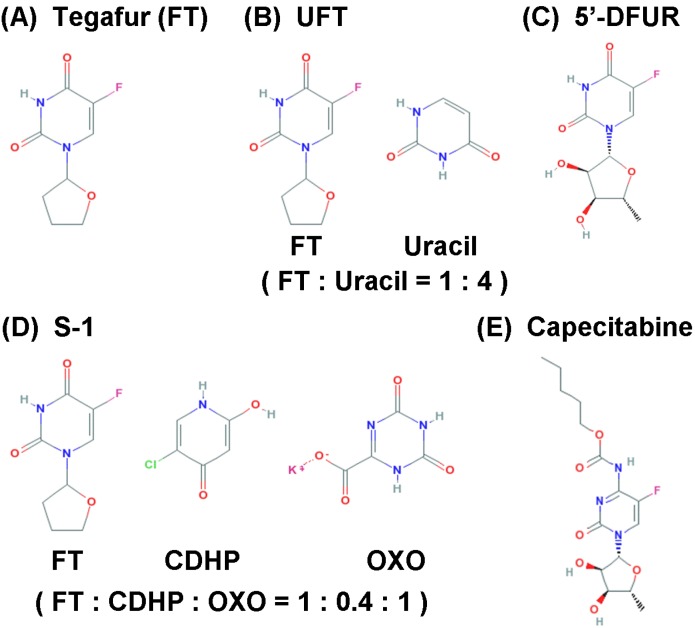
Structures of oral 5-FU drugs. (**A**) Tegafur; (**B**) UFT; (**C**) 5’-DFUR; (**D**) S-1; (**E**) Capecitabine.

As described in the “Introduction” section, 5-FU is a key anticancer drug for the treatment of various malignancies, and continuous 5-FU infusion regimens have been frequently used because of the apparent time-dependent effects of the drug. However, recent studies have shown that the continuous 5-FU infusion chemotherapies can be replaced with orally-administrable 5-FU drugs in some regimens, without any significant changes in either efficacy or adverse events [23,24]. In addition, oral administration of drugs allows several types of iatrogenic issues to be avoided. For the continuous infusion regimens such as FOLFOX or FOLFIRI, the implantation of a central venous port is required, but complications such as pneumothorax, hemothorax, or disconnection of the devices can occur. Furthermore, catheter-related infection or thrombosis is a serious problem for cancer patients [25,26]. The cost and benefit balance with the use of the central venous port system has been discussed [27], and recent clinical studies revealed that patients prefer oral administration rather than continuous infusion procedures. As such, orally-administered 5-FU regimens are likely to become more common in the clinical setting. Some fluoropyrimidines such as BOF-A2 (Emitefur) and Galocitabine (Ro 09-1390) are under development but not clinically available. In this section, we summarize the information currently available about orally-administrable 5-FU drugs (Table 1 and Figure 2). 

**Table 1:** Orally-administrable 5-FU drugs.

Drug name	Structure (Composition)	Concept	Developer	Refs.
Tegafur	1-(2-Tetrahydrofuryl)-5-fluorouracil	Prodrug	National Institute for Organic Syntheses (Latvia)	[28]
UFT	FT:Uracil = 1:4	Prodrug, DPD inhibitor	Osaka University (Japan)	[30]
5’-DFUR	5’-Deoxy-5-fluorouridine	Prodrug	Hoffmann-La Roche (Switzerland); Nippon Roche Research Center (Japan)	[38,39]
S-1	FT:CDHP:OXO = 1:0.4:1	DPD inhibitor, OPRT inhibitor	Taiho Pharmaceuticals (Japan)	[40]
Capecitabine	N4-Pentyloxycarbonyl-5′-deoxy-5-fluorocytidine	Prodrug	Nippon Roche Research Center (Japan)	[44]

### 3.1. Tegafur

1-(2-Tetrahydrofuryl)-5-fluorouracil (tegafur, FT, FT-207, Futrafur, Ftorafur, *etc*.) was developed as a 5-FU prodrug in the Soviet Union during the Cold War (as reported in 1967 by Giller *et al*. in a Russian record [28]). In 1970, the drug was introduced to Taiho Pharmaceuticals (Japan). Utilizing the benefits of FT, including: 1) its excellent absorbability from the GI tract and 2) its slight conversion to 5-FU in the GI tract, the development of orally-administrable FT was attempted, accomplished and reported in 1977 [29,30]. FT was shown to be gradually converted to 5-FU via cytochrome p450 enzymes in hepatic microsomes [31].

### 3.2. UFT

UFT consists of uracil and FT. Uracil competes with 5-FU for DPD activity [32,33], resulting in a prolonged 5-FU half-life. To optimize the molecular ratio of FT and uracil, Fujii *et al*., at the Institute for Protein Research (Osaka University, Japan), analyzed *in vivo* rat models administered with the combination of drugs, and revealed the optimal molar ratio to be 1:4 [34], which led to the introduction of UFT in 1985. In 1978, Fujii *et al*. also reported that the antitumor activity of FT on sarcoma-180 and AH-130 tumors was enhanced by oral administration of uracil, deoxyuridine or uridine [30], and this enhancement of the antitumor activity of FT increased with uracil, which caused a more extensive enhancement than did deoxyuridine or uridine. Furthermore, biochemical modulation of 5-FU had been investigated [35] using methotrexate, trimetrexate, interferon-α, leucovorin (LV) [36], and *N*-(phosphon-acetyl)-l-aspartic acid. The addition of LV to UFT regimens increases the available reduced folates, and thereby stabilizes the binding of FdUMP to TS, eventually inhibiting DNA synthesis. In 1997, Rustum *et al.* showed that LV increased the antitumor activity of UFT in the rat [32]; and Ichikura *et al.* showed that UFT with LV enhanced the inhibition of TS activity in gastric cancer patients [37]. In fact, the combination of 5-FU-based drugs with LV has been regarded as one of the standard treatments for colorectal cancer. These results eventually led to the development of S-1.

### 3.3. 5’-DFUR

In 1979, Cook *et al*. at Hoffmann-La Roche (Switzerland) [38] and Ishitsuka *et al*. in 1980 at the Nippon Roche Research Center (Japan) [39] reported the development of 5’-deoxy-5-fluorouridine (5’-DFUR, doxyfluridine, 5’-fluoro-5’-deoxyuridine, Ro 21-9738, Furtulon, *etc*.). The compound 5’-DFUR is parenterally and orally effective, and its activity was better than that of other fluorinated pyrimidines available at that time. A subline of L1210 leukemia cells was resistant to 5’-DFUR, and Ishitsuka *et al*. revealed that its resistance to 5’-DFUR was due to the lack of the UrdPase [39]. This is because 5’-DFUR is considered to be a depot form of 5-FU, which can be promptly activated by UrdPase [39]. Capecitabine (see below) was developed as the next generation of 5’-DFUR.

### 3.4. S-1

**Figure 3:**
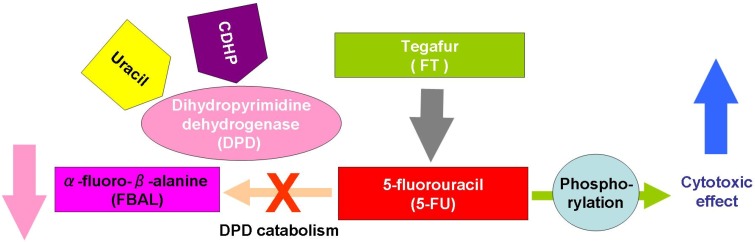
The metabolism of S-1.

After the development of UFT, Shirasaka *et al*. focused on the development of a novel oral FT-based fluoropyrimidine agent. They developed the next-generation drug, S-1, which both enhances the anticancer activity of 5-FU and reduces its GI toxicity [40]. The development of S-1 was based on two important findings: 1) 5-chloro-2,4-dihydroxypyridine (CDHP, Gimeracil, gimestat, *etc*.) is a DPD inhibitor, and 2) potassium oxonate (OXO) is an OPRT inhibitor (Figure 3).

Tatsumi *et al*. at Otsuka and Taiho Pharmaceuticals (Japan) investigated about 30 compounds for their inhibitory effects of DPD, mainly focusing on pyrimidines, barbituric acid and pyridine derivatives [41]; and in 1987 they reported that 3-cyano-2,6-dihydroxypyrimidine (CNDP) and CDHP were the strongest inhibitors of DPD [41]. Next, Shirasaka *et al*. [42] investigated the possibility of decreasing the GI toxicity of 5-FU without reducing its antitumor activity in rats. OXO localizes in the GI mucosa and selectively inhibits the OPRT, which inhibits 5-FU phosphorylation to FUMP, limiting GI toxicity effects (diarrhea, nausea and vomiting) [42]. In 1993, they reported that OXO inhibited the phosphorylation of 5-FU to FUMP catalyzed by pyrimidine phosphoribosyl-transferase, in a different manner from allopurinol. With experiments using Yoshida sarcoma-bearing rats, OXO was found to inhibit the formation of FUMP from 5-FU, with its subsequent incorporation into the RNA fractions of the small and large intestine, but not of the tumor and bone marrow tissues. This selective inhibition of 5-FU phosphorylation in the GI tract was due to the much higher concentrations of OXO in GI tissues than in other tissues and in the blood [42].

Based on these findings, CDHP and FT were simultaneously given orally to Yoshida sarcoma-bearing rats in various molar ratios, and then OXO was given orally during consecutive administration of the FT-CDHP mixture to find out the best condition to protect the animals from body weight loss without affecting the high antitumor efficacy of the FT-CDHP mixture [40]. Shirasaka *et al*. finally proposed a suitable formulation of the FT-based anticancer drug, called S-1, consisting of FT, CDHP and OXO at a 1:0.4:1 molar ratio and showed that it had tumor-selective cytotoxicity. S-1 is designed to reduce the GI toxicity of 5-FU; and in 2005 Muneoka *et al*. also reported that S-1 may be administered safely to patients with 5-FU-induced cardiotoxicity in whom FBAL is related to adverse events [43]. Recently, a combination granule version of S-1 has become commercially available.

### 3.5. Capecitabine

**Figure 4:**
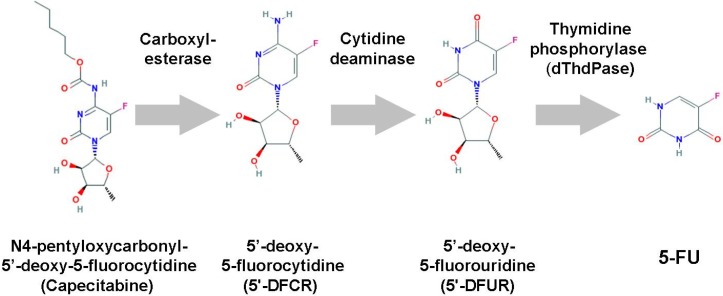
The metabolism of capecitabine.

Capecitabine (N4-pentyloxycarbonyl-5′-deoxy-5-fluorocytidine, Xeloda™, Ro 09-1978, *etc*.) is an oral fluoropyrimidine carbamate [44], which is selectively converted to 5-FU in tumors through a cascade of three enzymes: (1) carboxylesterase, which is almost exclusively located in the liver and hepatoma, but not in other tumors and normal tissues; (2) cytidine deaminase, which is located in the liver and various types of solid tumors, and 3) dThdPase, which is more concentrated in various types of tumor tissues than in normal tissues (Figure 4). 

Miwa *et al*. investigated the tissue locali zation of the three enzymes in humans [44], and these unique tissue locali zation patterns enabled the design of capecitabine. Oral capecitabine passes intact through the intestinal tract, but is converted first by carboxylesterase to 5’-deoxy-5-fluorocytidine (5'-DFCR) in the liver, then by cytidine deaminase to 5’-DFUR in the liver and tumor tissues, and finally by dThdPase to 5-FU in tumors. To design the optimized fluoropyrimidine carbamate, a series of N4-alkoxylcarbonyl derivatives were screened for hydrolysis to 5'-DFCR, specifically by carboxylesterase [45]. During the screening process, derivatives having an N4-alkoxylcarbonyl moiety with a C4-C6 alkyl chain were the most susceptible to human carboxylesterase, which led to the development of capecitabine. In 1998, Ishikawa *et al*. at the Nippon Roche Research Center investigated the efficacy of capecitabine and 5-FU in xenograft models implanted with human colon cancer cells [46]. Their results supported the notion that the inefficient conversion of 5’-DFUR to 5-FU by dThdPase in tumors would represent a mechanism of resistance. In contrast, even in tumors with sufficient levels of dThdPase, capecitabine was not effective if DPD levels were very high, and its efficacy was consequently found to be well-correlated with and dependent on the ratio of these two enzymes – dThdPase and DPD – in tumors [46]. The efficacy of capecitabine can be optimized by selecting patients who have tumors with a high ratio of dThdPase to DPD activities.

HFS is a cutaneous adverse event that occurs in some patients treated with fluoropyrimidines, which can severely disrupt the daily lives of patients. It is also a leading cause of interruption of capecitabine regimens as well [47]. In order to test the hypothesis that the occurrence of HFS could be related to tissue-specific expression of drug-metabolizing enzymes in the skin of the palms and soles, Milano *et al*. measured the expression of dThdPase (activation pathway), DPD (catabolic pathway) and cell proliferation (Ki67) in the skin of the palm (target tissue for HFS) and of the lower back (control area) with punch biopsy specimens [48]. Their study revealed that dThdPase and DPD expression levels were significantly greater in the palm relative to the back, and that dThdPase-facilitated local production of 5-FU in the palm during capecitabine treatment could explain the occurrence of HFS. In addition, the accumulated findings from clinical trials show the benefits of DPD inhibition on decreasing the risk of HFS [47].

The efficacy of co-administr ation of a series of DPD inhibitors with capecitabine has been investigated. A DPD inhibitor, RO0094889, which is a prodrug of 5-vinyluracil, was designed to generate 5-vinyluracil selectively in tumor tissues by sequential conversion by three enzymes responsible for the metabolism of capecitabine [49]. RO0094889 and various DPD inhibitors have been analyzed for co-administr ation with capecitabine. Nevertheless, HFS occurs more frequently with 5-FU delivered by continuous infusion [5] or with the 5-FU oral derivative capecitabine, ratherthan with bolus 5-FU therapy. 

## 4. Conclusions

Recently clinical studies on S-1 and capecitabine, comparing their efficacy and adverse events, have been reported, mainly from Korea [50,51]. The accumulated results will provide benefits that can optimize the treatment of cancer patients. The information obtained from the studies described in this review may give us better direction for the appropriate use of the oral 5-FU drugs. For example, the assessment of the dThdPase and DPD levels may provide evidence of patients who would be good/poor responders to therapy. Patients with low dThdPase activity and inefficient conversion of 5’-DFUR to 5-FU, may present resistance to capecitabine. The activities of carboxylesterase and cytidine deaminase may also affect the efficacy of capecitabine. Among patients with high DPD activity, S-1 may exhibit better efficacy; on the other hand, capecitabine may show more powerful effects along with DPD inhibitors in tumor cells. Although recent studies support the notion that the continuous 5-FU infusion chemotherapies can be replaced with orally-administrable 5-FU drugs in some regimens, it will be necessary for us to remember that the metabolism of orally-administered 5-FU differs from that of infusional 5-FU, because orally-administered 5-FU undergoes more diverse metabolism in the gastrointestinal tract and in the liver, with various enzymes. On the other hand, it is essential to elucidate the pharmacokinetic mechanism of each of the newly-developed drugs, to ensure the selection of the proper drug(s) for each patient in the clinical setting, and to further develop the optimized drug derivatives. This will require the collaboration of clinicians, molecular biologists and preclinical drug researchers.

## References

[1] Heidelberger C., Chaudhuri N.K., Danneberg P., Mooren D., Griesbach L., Duschinsky R., Schnitzer R.J., Pleven E., Scheiner J. (1957). Fluorinated pyrimidines, a new class of tumour-inhibitory compounds. Nature.

[2] Rutman R.J., Cantarow A., Paschkis K.E. (1954). The catabolism of uracil *in vivo* and *in vitro*. J. Biol. Chem..

[3] Handschumacher R.E., Welch A.D. (1956). Microbial studies of 6-azauracil, an antagonist of uracil. Cancer Res..

[4] Skipper H.E., Schabel F.M., Wilcox W.S. (1964). Experimental evaluation of potential anticancer agents. XIII. On the criteria and kinetics associated with "curability" of experimental leukemia. Cancer Chemother. Rep..

[5] Meta-analysis Group In Cancer (1998). Efficacy of intravenous continuous infusion of fluorouracil compared with bolus administration in advanced colorectal cancer. J. Clin. Oncol..

[6] Saif M.W., Syrigos K.N., Katirtzoglou N.A. (2009). S-1: A promising new oral fluoropyrimidine derivative. Expert Opin. Investig. Drugs.

[7] Wohlhueter R.M., McIvor R.S., Plagemann P.G. (1980). Facilitated transport of uracil and 5-fluorouracil, and permeation of orotic acid into cultured mammalian cells. J. Cell. Physiol..

[8] Longley D.B., Harkin D.P., Johnston P.G. (2003). 5-fluorouracil: mechanisms of action and clinical strategies. Nat. Rev. Cancer.

[9] Houghton J.A., Houghton P.J., Wooten R.S. (1979). Mechanism of induction of gastrointestinal toxicity in the mouse by 5-fluorouracil, 5-fluorouridine, and 5-fluoro-2'-deoxyuridine. Cancer Res..

[10] Schuetz J.D., Wallace H.J., Diasio R.B. (1984). 5-fluorouracil incorporation into DNA of CF-1 mouse bone marrow cells as a possible mechanism of toxicity. Cancer Res..

[11] Meta-Analysis Group In Cancer (1998). Toxicity of fluorouracil in patients with advanced colorectal cancer: effect of administration schedule and prognostic factors. J. Clin. Oncol..

[12] Diasio R.B., Harris B.E. (1989). Clinical pharmacology of 5-fluorouracil. Clin. Pharmacokinet..

[13] Heggie G.D., Sommadossi J.P., Cross D.S., Huster W.J., Diasio R.B. (1987). Clinical pharmaco-kinetics of 5-fluorouracil and its metabolites in plasma, urine, and bile. Cancer Res..

[14] Koenig H., Patel A. (1970). Biochemical basis for fluorouracil neurotoxicity. The role of Krebs cycle inhibition by fluoroacetate. Arch. Neurol..

[15] Okeda R., Shibutani M., Matsuo T., Kuroiwa T., Shimokawa R., Tajima T. (1990). Experimental neurotoxicity of 5-fluorouracil and its derivatives is due to poisoning by the monofluorinated organic metabolites, monofluoroacetic acid and alpha-fluoro-beta-alanine. Acta Neuropathol..

[16] Matsubara I., Kamiya J., Imai S. (1980). Cardiotoxic effects of 5-fluorouracil in the guinea pig. Jpn. J. Pharmacol..

[17] Santi D.V., McHenry C.S. (1972). 5-Fluoro-2'-deoxyuridylate: covalent complex with thymidylate synthetase. Proc. Natl. Acad. Sci. USA.

[18] Jackson R.C., Grindley G.B., Sirotnak F.M., Burchell J.J., Ensminger W.D. (1984). The biochemical basis for methotrexate cytotoxicity. Folate Antagonists as Therapeutic Agents.

[19] Yoshioka A., Tanaka S., Hiraoka O., Koyama Y., Hirota Y., Ayusawa D., Seno T., Garrett C., Wataya Y. (1987). Deoxyribonucleoside triphosphate imbalance. 5-Fluorodeoxyuridine-induced DNA double strand breaks in mouse FM3A cells and the mechanism of cell death. J. Biol. Chem..

[20] Mitrovski B., Pressacco J., Mandelbaum S., Erlichman C. (1994). Biochemical effects of folate-based inhibitors of thymidylate synthase in MGH-U1 cells. Cancer Chemother. Pharmacol..

[21] Grem J.L., Fischer P.H. (1989). Enhancement of 5-fluorouracil's anticancer activity by dipyridamole. Pharmacol. Ther..

[22] Showalter S.L., Showalter T.N., Witkiewicz A., Havens R., Kennedy E.P., Hucl T., Kern S.E., Yeo C.J., Brody J.R. (2008). Evaluating the drug-target relationship between thymidylate synthase expression and tumor response to 5-fluorouracil. Is it time to move forward?. Cancer Biol. Ther..

[23] Lembersky B.C., Wieand H.S., Petrelli N.J., O'Connell M.J., Colangelo L.H., Smith R.E., Seay T.E., Giguere J.K., Marshall M.E., Jacobs A.D. (2006). Oral uracil and tegafur plus leucovorin compared with intravenous fluorouracil and leucovorin in stage II and III carcinoma of the colon: results from National Surgical Adjuvant Breast and Bowel Project Protocol C-06. J. Clin. Oncol..

[24] Boku N., Yamamoto S., Fukuda H., Shirao K., Doi T., Sawaki A., Koizumi W., Saito H., Yamaguchi K., Takiuchi H. (2009). Fluorouracil *versus* combination of irinotecan plus cisplatin *versus* S-1 in metastatic gastric cancer: A randomised phase 3 study. Lancet Oncol..

[25] Mansfield P.F., Hohn D.C., Fornage B.D., Gregurich M.A., Ota D.M. (1994). Complications and failures of subclavian-vein catheterization. N. Engl. J. Med..

[26] Agnelli G., Verso M. (2006). Therapy Insight: venous-catheter-related thrombosis in cancer patients. Nat. Clin. Pract. Oncol..

[27] Lokich J.J., Moore C.L., Anderson N.R. (1996). Comparison of costs for infusion *versus* bolus chemotherapy administration–Part two. Use of charges *versus* reimbursement for cost basis. Cancer.

[28] Giller S.A., Zhuk R.A., Lidak M.Iu. (1967). Analogs of pyrimidine nucleosides. I. N1-(alpha-furanidyl) derivatives of natural pyrimidine bases and their antimetabolities. Dokl. Akad. Nauk. SSSR..

[29] Toide H., Akiyoshi H., Minato Y., Okuda H., Fujii S. (1977). Comparative studies on the metabolism of 2-(tetrahydrofuryl)-5-fluorouracil and 5-fluorouracil. Gann.

[30] Fujii S., Ikenaka K., Fukushima M., Shirasaka T. (1978). Effect of uracil and its derivatives on antitumor activity of 5-fluorouracil and 1-(2-tetrahydrofuryl)-5-fluorouracil. Gann.

[31] El Sayed Y.M., Sadée W. (1983). Metabolic activation of R,S-1-(tetrahydro-2-furanyl)-5-fluorouracil (ftorafur) to 5-fluorouracil by soluble enzymes. Cancer Res..

[32] Rustum Y.M. (1997). Mechanism-based improvement in the therapeutic selectivity of 5-FU prodrug alone and under conditions of metabolic modulation. Oncology.

[33] Diasio R.B. (1998). The role of dihydropyrimidine dehydrogenase (DPD) modulation in 5-FU pharmacology. Oncology.

[34] Fujii S., Kitano S., Ikenaka K., Shirasaka T. (1979). Effect of coadministration of uracil or cytosine on the anti-tumor activity of clinical doses of 1-(2-tetrahydrofuryl)-5-fluorouracil and level of 5-fluorouracil in rodents. Gann.

[35] Hoff P.M., Cassidy J., Schmoll H.J. (2001). The evolution of fluoropyrimidine therapy: From intravenous to oral. Oncologist.

[36] Poon M.A., O'Connell M.J., Wieand H.S., Krook J.E., Gerstner J.B., Tschetter L.K., Levitt R., Kardinal C.G., Mailliard J.A. (1991). Biochemical modulation of fluorouracil with leucovorin: confirmatory evidence of improved therapeutic efficacy in advanced colorectal cancer. J. Clin. Oncol..

[37] Ichikura T., Tomimatsu S., Okusa Y., Yahara T., Uefuji K., Tamakuma S. (1996). Thymidylate synthase inhibition by an oral regimen consisting of tegafur-uracil (UFT) and low-dose leucovorin for patients with gastric cancer. Cancer Chemother. Pharmacol..

[38] Cook A.F., Holman M.J., Kramer M.J., Trown P.W. (1979). Fluorinated pyrimidine nucleosides. 3. Synthesis and antitumor activity of a series of 5'-deoxy-5-fluoropyrimidine nucleosides. J. Med. Chem..

[39] Ishitsuka H., Miwa M., Takemoto K., Fukuoka K., Itoga A., Maruyama HB. (1980). Role of uridine phosphorylase for antitumor activity of 5'-deoxy-5-fluorouridine. Gann.

[40] Shirasaka T., Shimamato Y., Ohshimo H., Yamaguchi M., Kato T., Yonekura K., Fukushima M. (1996). Development of a novel form of an oral 5-fluorouracil derivative (S-1) directed to the potentiation of the tumor selective cytotoxicity of 5-fluorouracil by two biochemical modulators. Anticancer Drugs.

[41] Tatsumi K., Fukushima M., Shirasaka T., Fujii S. (1987). Inhibitory effects of pyrimidine, barbituric acid and pyridine derivatives on 5-fluorouracil degradation in rat liver extracts. Jpn. J. Cancer Res..

[42] Shirasaka T., Shimamoto Y., Fukushima M. (1993). Inhibition by oxonic acid of gastrointestinal toxicity of 5-fluorouracil without loss of its antitumor activity in rats. Cancer Res..

[43] Muneoka K., Shirai Y., Yokoyama N., Wakai T., Hatakeyama K. (2005). 5-Fluorouracil cardiotoxicity induced by alpha-fluoro-beta-alanine. Int. J. Clin. Oncol..

[44] Miwa M., Ura M., Nishida M., Sawada N., Ishikawa T., Mori K., Shimma N., Umeda I., Ishitsuka H. (1998). Design of a novel oral fluoropyrimidine carbamate, capecitabine, which generates 5-fluorouracil selectively in tumours by enzymes concentrated in human liver and cancer tissue. Eur. J. Cancer.

[45] Shimma N., Umeda I., Arasaki M., Murasaki C., Masubuchi K., Kohchi Y., Miwa M., Ura M., Sawada N., Tahara H. (2000). The design and synthesis of a new tumor-selective fluoropyrimidine carbamate, capecitabine. Bioorg. Med. Chem..

[46] Ishikawa T., Utoh M., Sawada N., Nishida M., Fukase Y., Sekiguchi F., Ishitsuka H. (1998). Tumor selective delivery of 5-fluorouracil by capecitabine, a new oral fluoropyrimidine carbamate, in human cancer xenografts. Biochem. Pharmacol..

[47] Yen-Revollo J.L., Goldberg R.M., McLeod H.L. (2008). Can inhibiting dihydropyrimidine dehydrogenase limit hand-foot syndrome caused by fluoropyrimidines?. Clin. Cancer Res..

[48] Milano G., Etienne-Grimaldi M.C., Mari M., Lassalle S., Formento J.L., Francoual M., Lacour J.P., Hofman P. (2008). Candidate mechanisms for capecitabine-related hand-foot syndrome. Br. J. Clin. Pharmacol..

[49] Hattori K., Kohchi Y., Oikawa N., Suda H., Ura M., Ishikawa T., Miwa M., Endoh M., Eda H., Tanimura H. (2003). Design and synthesis of the tumor-activated prodrug of dihydropyrimidine dehydrogenase (DPD) inhibitor, RO0094889 for combination therapy with capecitabine. Bioorg. Med. Chem. Lett..

[50] Lee J.L., Kang Y.K., Kang H.J., Lee K.H., Zang D.Y., Ryoo B.Y., Kim J.G., Park S.R., Kang W.K., Shin D.B. (2008). A randomised multicentre phase II trial of capecitabine vs S-1 as first-line treatment in elderly patients with metastatic or recurrent unresectable gastric cancer. Br. J. Cancer.

[51] Seol Y.M., Song M.K., Choi Y.J., Kim G.H., Shin H.J., Song G.A., Chung J.S., Cho G.J. (2009). Oral fluoropyrimidines (capecitabine or S-1) and cisplatin as first line treatment in elderly patients with advanced gastric cancer: a retrospective study. Jpn. J. Clin. Oncol..

